# Effects of Resveratrol Derivatives on Melanogenesis and Antioxidant Activity in B16F10 Cells

**DOI:** 10.3390/ijms26114999

**Published:** 2025-05-22

**Authors:** Soyeon Kim, Changho Jhin, Sullim Lee, Ho Sik Rho, Chan Yoon Park

**Affiliations:** 1 Department of Food and Nutrition, The University of Suwon, Hwaseong 18323, Republic of Korea; 2 Research and Development Department, 1778 LivingTech Co., Ltd., Sejong 30084, Republic of Korea; 3 Department of Life Science, College of Bio-Nano Technology, Gachon University, Seongnam 13120, Republic of Korea; 4 Department of Chemical Engineering, The University of Suwon, Hwaseong 18323, Republic of Korea

**Keywords:** melanogenesis, resveratrol, dihydroresveratrol, resveratrol derivative, tyrosinase, antioxidant effect

## Abstract

Excessive melanogenesis causes abnormal pigmentation and a higher risk of skin disorders (e.g., melanoma). Resveratrol (RSV), a natural polyphenol, exerts antioxidant and anti-aging effects. However, the effects of RSV and its derivatives on melanogenesis remain unclear. This study investigated their effects on melanogenesis and antioxidant activity in B16F10 cells. After measuring cell viability, B16F10 cells were incubated with 50 µM of RSV, dihydroresveratrol (DIRSV), and other RSV derivatives for 24 h. The relative melanin content and tyrosinase activity were quantified. The protein and mRNA levels of melanogenesis-related genes (MITF, CREB, TYR, and TRP) and the binding affinity of RSV derivatives to their target proteins were measured. The antioxidant activity was evaluated using ABTS and DPPH assays. RSV and DIRSV (50 µM) significantly reduced melanin content and tyrosinase activity, respectively. However, other derivatives had no significant effects. RSV, DIRSV, and other derivatives significantly suppressed MITF and CREB levels. Additionally, DIRSV significantly reduced p-CREB and TYR protein levels and showed a higher affinity for CREB than RSV, despite no significant changes in *MITF*, *TYR*, or *TRP* mRNA levels. In the antioxidant assays, RSV and DIRSV exhibited significant ABTS and DPPH radical scavenging activities. DIRSV, like RSV, inhibits melanogenesis and exhibits antioxidant effects in B16F10 cells. However, RSV derivatives demonstrate partial antioxidant activity and inhibit melanogenesis-related proteins but do not significantly affect melanogenesis. DIRSV’s practical applications as a skin-protective and -whitening agent warrant further exploration.

## 1. Introduction

Melanin, a heteropolymer comprising 5,6-dihydroxyindole and 5,6-dihydroxyindole-2-carboxylic acid, is synthesized by epidermal melanocytes. It serves as a crucial pigment that is stored in the basal layer of epidermal cells and determines hair, eye, and skin color [1]. Melanin plays an essential role in protecting the skin from damage and inflammation induced by ultraviolet (UV) radiation and external stimuli, significantly contributing to the maintenance of skin health [2]. However, disruptions in melanin homeostasis or its excessive production can result in various skin problems, including abnormal pigmentation, freckles, wrinkles, and photoaging. Such conditions may also aggravate skin inflammation and heighten the risk of skin cancer [3].

Melanogenesis, the process of melanin synthesis, commences with interaction between the melanocortin 1 receptor (MC1R), located on the melanocyte membrane, and its agonists: melanocyte-stimulating hormone (α-MSH), adrenocorticotropic hormone, and other hormones [4]. This interaction activates adenylyl cyclase, thereby increasing the intracellular levels of cyclic adenosine monophosphate (cAMP), which in turn activates protein kinase A (PKA). Activated PKA phosphorylates the cAMP response element-binding protein (CREB), promoting the expression of microphthalmia-associated transcription factor (MITF) [5]. MITF is a transcription factor that regulates the expression of key melanin-synthesizing enzymes, including tyrosinase, tyrosinase-related protein (TRP)-1, and TRP-2 [6]. Tyrosinase, the rate-limiting enzyme of melanogenesis, oxidizes tyrosine to dopa, and dopa to dopaquinone, and its activity correlates with the degree of melanin production [4]. With growing interest in skin whitening through the inhibition of melanin synthesis, research has increasingly focused on identifying active substances and food components, such as kojic acid and hydroquinone, that inhibit tyrosinase activity [7,8,9,10].

Oxidative stress is closely associated with melanin synthesis. It can be induced by UV exposure or H_2_O_2_ treatment, which reportedly increases melanin synthesis and pigmentation [11]. Therefore, oxidative stress, mediated by the generation of reactive oxygen species (ROS), can contribute to excessive melanin production. Additionally, the melanin synthetic pathway produces diverse ROS from both exogenous (e.g., UV exposure and inflammation) and endogenous sources during melanogenesis [12]. Since the catalytic activity of tyrosinase leads to the generation of O_2_^−^ and conversion of indole into quinone, both of which potentially induce oxidative stress in melanocytes, protective mechanisms are activated to counteract this stress [13,14]. For example, α-MSH binds to and activates MC1R, which in sequence increases the protein levels and activity of catalase, as well as antioxidant genes, including heme oxygenase-1 (HO-1), ferritin, and peroxiredoxin-1 [15]. The nuclear erythroid 2-related factor (Nrf2)–antioxidant response element (ARE) pathway reportedly protects against oxidative stress-induced cellular damage. Increased Nrf2 pathway activity enhances cellular resistance to oxidative stress induced by UV radiation and chemical substances [16,17,18]. Therefore, reducing oxidative stress is related to the inhibition of melanogenesis, which has led to growing interest in developing skin-whitening agents derived from natural substances that are known for their antioxidant properties.

Resveratrol (RSV) is a polyphenolic phytoalexin that is abundantly present in foods such as grapes, red wine, and berries; it exhibits multiple physiological activities (e.g., antioxidant, anticancer, cardiovascular protective, anti-inflammatory, and anti-aging effects) [19,20,21,22,23]. In addition, several studies have reported that RSV can inhibit tyrosinase, TRP-1, and TRP-2 activity and reduce melanin production, highlighting its potential as a skin-whitening agent [11,24,25]. However, high-concentration (>50 µM) RSV treatment decreases B16F10 melanoma cell viability [26]. Therefore, researchers are actively working to develop RSV formulations that retain its beneficial effects while minimizing its effect on cell viability. Structurally, RSV is a compound in which two phenyl groups (A ring and B ring) are connected by a double bond. The A ring has hydroxyl groups at positions 2 and 4, and the B ring has a hydroxyl group at position 4. It is known that the various biological activities of RSV are due to the three hydroxyl groups [27]. But RSV also presents a limitation due to its poor photostability, which restricts its practical applications. RSV exists in two isomeric forms, cis and trans, of which the trans isomer is known to be more stable and biologically active. Under various UV irradiation conditions, trans-RSV readily isomerizes to cis-RSV, which is considered relatively less stable and biologically less active [28]. Therefore, identifying a photostable alternative to trans-RSV is needed. There are two ways to obtain photostable RSV alternatives. One is to eliminate the double bond, and the other is to replace the double bond with a chemically stable linker. Several RSV derivatives and metabolites have been investigated for their potential to downregulate melanin synthesis [18,29,30,31,32]. Oxyresveratrol, an RSV derivative with a hydroxyl group (–OH) added to its structure, possesses a 2,3,5,4′-tetrahydroxystilbene structure and reportedly inhibits pigmentation by suppressing tyrosinase activity [18]. Dihydroresveratrol (DIRSV) has also been reported to inhibit the activity of tyrosinase-related enzymes, including TRP-1 and TRP-2 [29].

In this study, we synthesized four trans-RSV derivatives (RHS-0139, RHS-0140, RHS-0141, and RHS-0142) that contain an amide linker by fixing the 2,4 hydroxyl groups of the A ring and modifying the hydroxyl group of the B ring and produced DIRSV by reducing the double bond of trans-RSV. By comparing the biological activity of RSV and its derivatives, we aim to identify pharmacophores in the structure of RSV (double bonds and hydroxyl groups in A and B ring). Trans-RSV and five of its derivatives—DIRSV, RHS-0139, RHS-0140, RHS-0141, and RHS-0142—were evaluated for their effects on melanogenesis in B16F10 melanoma cells without impacting the cell viability. The expression levels and activity of the melanogenesis-related proteins were assessed after treatment with these derivatives. Additionally, the binding affinities of these derivatives for melanogenesis-related proteins and their antioxidant capacities were evaluated to determine their potential as candidates for modulating melanogenesis.

## 2. Results

**Figure 1:**
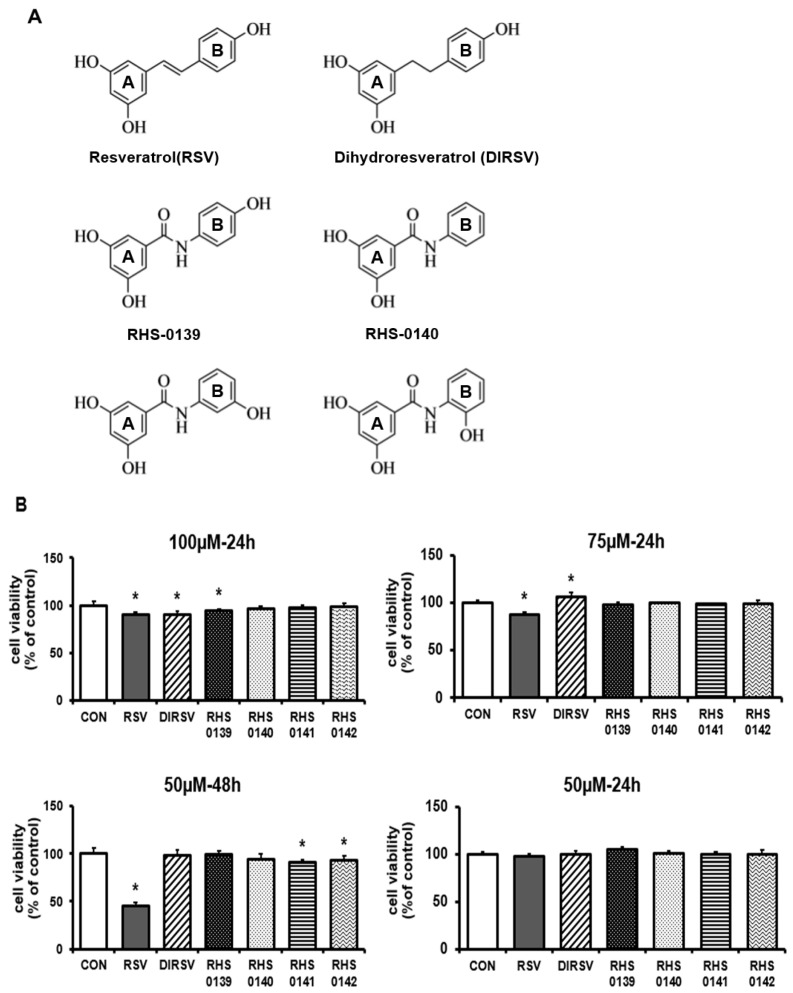
(**A**) Structures of resveratrol and its derivatives. Trans-resveratrol (RSV); Dihydroresveratrol (DIRSV); RHS-0139; RHS-0140; RHS-0141; RHS-0142. (**B**) Effects of RSV, DIRSV, RHS-0139, RHS-0140, RHS-0141, and RHS-0142 on B16F10 cell viability. Results are expressed as the average of three independent experiments, and data are expressed as the mean ± SE (* *p* < 0.05).

The structures of trans-RSV and its derivatives (DIRSV, RHS-0139, RHS-0140, RHS-0141, and RHS-0142) are shown in Figure 1A, and their exact molecular structures were also characterized (Figure S1). MTT assays were conducted to evaluate cell viability following treatment with RSV and its derivatives (Figure 1B). Treatment with RSV, DIRSV, and RHS-0139 at 100 μM for 24 h significantly reduced the cell viability. At 75 μM for 24 h, only RSV decreased the cell viability (by 13%), whereas DIRSV increased it; nonetheless, the other derivatives yielded no significant difference. No significant difference in cell viability was noted in the “50 μM 24 h” treatment group for all compounds. However, RSV treatment at 50 μM for 48 h caused a significant reduction in cell viability compared with 24 h treatment at the same concentration. Based on these results, subsequent experiments were performed using 24 h treatment at 50 μM.

**Figure 2:**
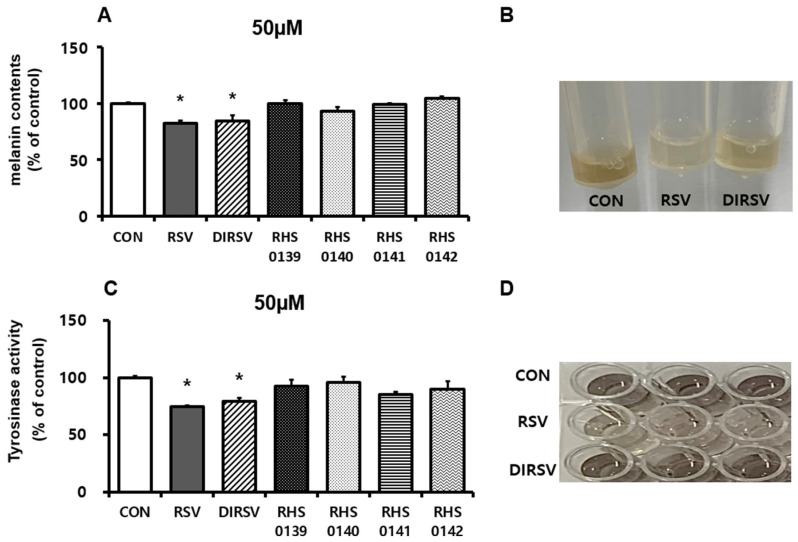
(**A**) Effects of resveratrol (RSV), dihydroresveratrol (DIRSV), RHS-0139, RHS-0140, RHS-0141, and RHS-0142 (50 μM) on melanogenesis in B16F10 cells. (**B**) Image of melanin extracted from B16F10 melanoma cells. (**C**) Effects of 24 treatment with RSV, DIRSV, RHS-0139, RHS-0140, RHS-0141, and RHS-0142 (50 μM) on tyrosinase activity in B16F10 cell. (**D**) Images of tyrosinase activity from B16F10 melanoma cell. Relative melanin content and tyrosinase activity were quantified using spectrophotometry and normalized to protein content. Results are averages of three independent experiments, and data are expressed as mean ± SE (* *p* < 0.05).

The effects of RSV and its derivatives on melanin synthesis in B16F10 cells were evaluated by measuring the melanin content and tyrosinase activity (Figure 2A–D). RSV and DIRSV treatment reduced the melanin content by approximately 18% and 15%, respectively, compared with the control (Figure 2A). A noticeable decrease in melanin content was observed in both the RSV and DIRSV treatment groups (Figure 2B). In contrast, no significant reduction in melanin content was observed with the other derivatives (RHS-0139, RHS-0140, RHS-0141, and RHS-0142).

Tyrosinase, a key oxidative enzyme in melanogenesis, serves a critical role in regulating melanin synthesis. To assess the effects of RSV and its derivatives on enzyme activity, the tyrosinase activity was measured. Consistent with the results for melanin content, RSV and DIRSV significantly inhibited tyrosinase activity by 26% and 21%, respectively, compared with the control (Figure 2C). In contrast, the other derivatives exerted no significant effect on tyrosinase activity.

**Figure 3:**
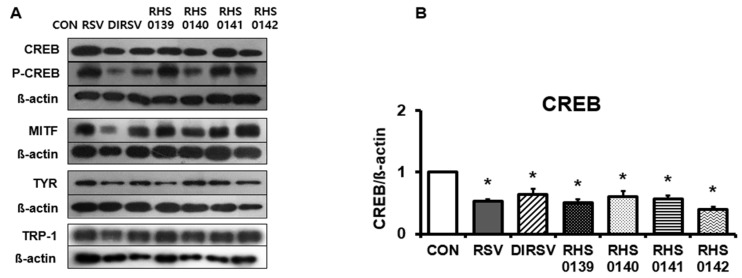

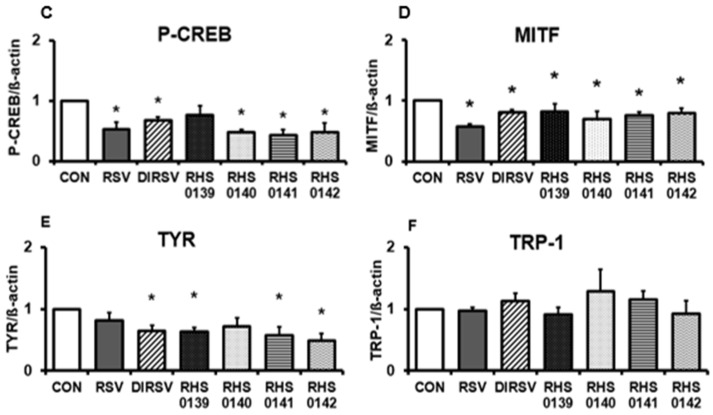
Protein levels of melanogenesis-related genes in B16F10 Cells. (**A**) Densitometry results of CREB, p-CREB, MITF, TYR, TRP-1, and β-actin. (**B**) CREB, (**C**) p-CREB, (**D**) MITF, (**E**) TYR, (**F**) TRP-1. B16F10 cells were treated with each sample (50 μM) for 24 h. Results are presented as average of three independent experiments, and data are expressed as mean ± SE (* *p* < 0.05).

Thereafter, the protein expression levels of MITF, TYR, TRP-1, CREB, and p-CREB, which are associated with the melanin synthesis pathway, were investigated following treatment with RSV and its derivatives in B16F10 cells (Figure 3). MITF is a well-established key transcription factor that regulates the transcription of melanogenesis-related genes. MITF activation commences with the phosphorylation of CREB, which translocates to the nucleus and binds to the MITF promoter, thereby promoting MITF expression. Activated MITF subsequently induces the transcription of melanin synthetase genes, such as tyrosinase, TRP-1, and TRP-2, playing an indispensable role in melanin biosynthesis and skin pigmentation. In the present study, RSV (*p* < 0.001), DIRSV (*p* = 0.004), RHS-0139 (*p* < 0.001), RHS-0140 (*p* = 0.002), RHS-0141 (*p* < 0.001), and RHS-0142 (*p* < 0.001) significantly downregulated CREB levels (Figure 3B). Similarly, RSV (*p* = 0.005), DIRSV (*p* = 0.041), RHS-0140 (*p* = 0.003), RHS-0141 (*p* = 0.001), and RHS-0142 (*p* = 0.003) significantly decreased p-CREB levels (Figure 3C). The MITF protein levels were also significantly decreased by RSV and all its derivatives (all *p* < 0.05) (Figure 3D). Regarding TYR expression, only DIRSV (*p* = 0.03), RHS-0139 (*p* = 0.04), RHS-0141 (*p* = 0.04), and RHS-0142 (*p* = 0.01) exhibited significant reductions (Figure 3E). In contrast, TRP-1 expression was not significantly affected by RSV or any of its derivatives (Figure 3F).

Based on the protein expression results, the binding affinities of RSV and its derivatives for melanin synthesis-related proteins were examined using in silico molecular docking (Table 1). For tyrosinase, RSV exhibited the strongest binding affinity (−7.3 kcal/mol). DIRSV yielded a slightly lower binding affinity (−7.0 kcal/mol), while RHS-0139, RHS-0140, RHS-0141, and RHS-0142 exhibited binding affinities of −7.1, −7.1, −7.0, and −7.1 kcal/mol, respectively, which were comparable to that of RSV. The binding affinity between tropolone, a known tyrosinase antagonist, and tyrosinase was determined to be −5.9 kcal/mol. In comparison, RSV and its derivatives exhibited significantly higher binding affinities for tyrosinase. In the case of TRP-1, RSV and its derivatives exhibited higher binding affinities than mimosine (−6.2 kcal/mol). Among them, RHS-0141 yielded the highest binding affinity (−7.7 kcal/mol), slightly exceeding the binding affinities of RSV (−7.5 kcal/mol) and DIRSV (−7.2 kcal/mol). Meanwhile, the small-molecule inhibitors ML329 and KG-501, which are known to directly bind to MITF and CREB, respectively, and inhibit their function as transcription factors [33,34], exhibited higher binding affinities for MITF and CREB compared to RSV and its derivatives. Among the RSV derivatives, DIRSV showed a slightly stronger binding affinity for CREB (−5.3 kcal/mol) than RSV (−5.2 kcal/mol), while RHS-0139, RHS-0140, RHS-0141, and RHS-0142 exhibited comparable affinities, ranging from −5.1 to −5.3 kcal/mol. For MITF, DIRSV exhibited a slightly lower binding affinity (−4.9 kcal/mol) than RSV (−5.1 kcal/mol) (Table 1).

**Figure 4:**
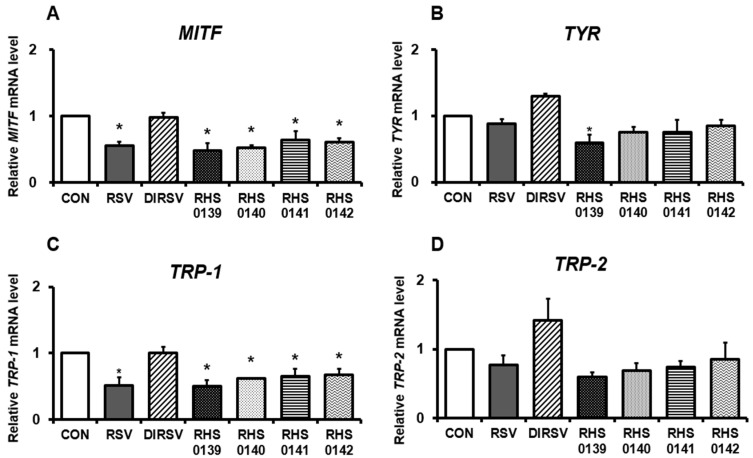
The mRNA expression levels of melanogenesis-related genes in B16F10 cells. (**A**) *MITF* (**B**) *TYR* (**C**) *TRP-1*, and (**D**) *TRP-2.* B16F10 cells were treated with each sample (50 μM) for 24 h. Results are presented as the average of three independent experiments, and data are expressed as the mean ± SE (* *p* < 0.05).

Notwithstanding, the mRNA expression levels of melanogenesis-associated proteins in B16F10 cells after treatment with RSV and its derivatives exhibited different expression patterns from the protein levels (Figure 4). Treatment with RSV, RHS-0139, RHS-0140, RHS-0141, and RHS-0142 significantly reduced the mRNA expression levels of *MITF* and *TRP-1* (all *p* < 0.05). However, no significant change in *MITF* or *TRP-1* expression was observed with DIRSV treatment. Only RHS-0139 treatment significantly reduced *TYR* expression (*p* = 0.01), with the other derivatives exerting no significant effects. The mRNA expression of TRP-2 was not significantly affected by RSV or any of its derivatives.

**Figure 5:**
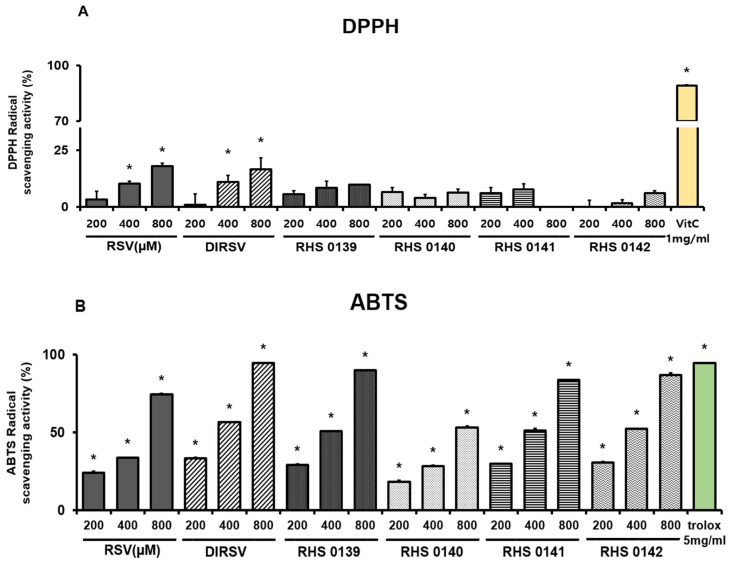
The antioxidant activities of resveratrol (RSV), dihydroresveratrol (DIRSV), RHS-0139, RHS-0140, RHS-0141, and RHS-0142. (**A**) DPPH and (**B**) ABTS assays. Results are presented as the average of three independent experiments, and data are expressed as the mean ± SE (* *p* < 0.05).

Since ROS overexpression and the resulting oxidative stress reportedly promote excessive melanin production, the antioxidant capabilities of RSV and its derivatives were evaluated. RSV, DIRSV, RHS-0139, RHS-0140, RHS-0141, and RHS-0142 were assessed for their ROS scavenging activities using the DPPH and ABTS assays (Figure 5). In both the DPPH and ABTS assays, RSV and DIRSV demonstrated significant ROS scavenging activity in a concentration-dependent manner. In the DPPH assay, at 400 and 800 μM, RSV exhibited scavenging activities of approximately 10% (*p* = 0.04) and 17% (*p* < 0.001), while DIRSV yielded activities of 11% (*p* = 0.03) and 16% (*p* = 0.002), respectively. Conversely, the other derivatives (RHS-0139, RHS-0140, RHS-0141, and RHS-0142) did not exhibit radical scavenging activities in the DPPH assay (Figure 5A). In the ABTS assay, RSV and DIRSV displayed high activity at concentrations of 400 and 800 μM, respectively, with DIRSV exhibiting scavenging activity of approximately 95% at 800 μM. The derivatives demonstrated higher scavenging activities than RSV at 800 μM, and notable results were observed for RHS-0140 (400 μM) and RHS-0142 (200 μM), which exhibited RSV-like activity levels at 400 μM (Figure 5B). Interestingly, at 800 μM, DIRSV displayed greater antioxidant activity than Trolox (5 mg/mL), underscoring its strong free radical scavenging ability (*p* < 0.001).

**Figure 6:**
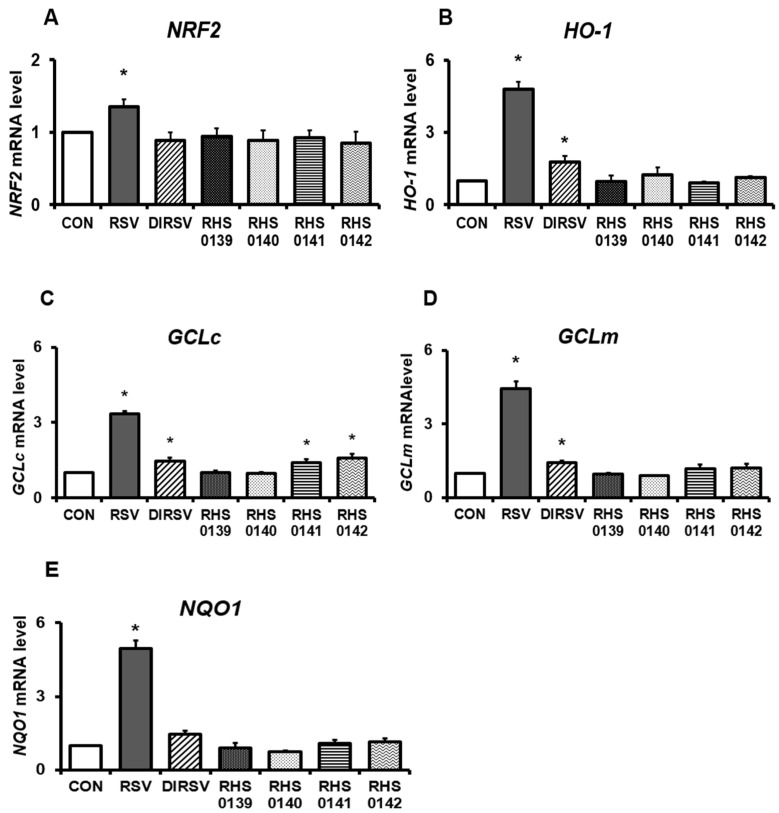
The mRNA expression levels of antioxidant effect-related genes in B16F10 cells. (**A**) *NRF2*, (**B**) *HO-1*, (**C**) *GCLc*, (**D**) *GCLm*, and (**E**) *NQO1*. B16F10 cells were treated with each sample (50 μM) for 24 h. Results are presented as the average of three independent experiments, and data are expressed as the mean ± SE (* *p* < 0.05).

We also investigated whether the mRNA expression levels of antioxidant-related markers are regulated by RSV and its derivatives in B16F10 cells (Figure 6). RSV treatment significantly increased the mRNA expression levels of *NRF2*, *HO-1*, *Glutamate–cysteine ligase catalytic subunit* (*GCLc*), *Glutamate-cysteine ligase regulatory subunit* (*GCLm*), and *NADH dehydrogenase 1* (*NQO1*) compared with the control. Likewise, DIRSV treatment significantly increased *HO-1*, *GCLc*, and *GCLm* mRNA expression levels (all *p* <0.05) and tended to increase *NQO1* expression, but it did not affect *NRF2* expression. Meanwhile, four of the derivatives (RHS-0139, RHS-0140, RHS-0141, and RHS-0142) did not yield significant differences in the mRNA expression levels of *NRF2*, *HO-1*, *GCLm*, and *NQO1*. However, RHS-0141 and RHS-0142 significantly increased the mRNA expression levels of *GCLc*.

## 3. Discussion

In this study, we reconfirmed the inhibitory effect of RSV on melanogenesis, as reported in previous studies [24,25], and examined whether its derivatives can suppress melanogenesis without exerting the cytotoxic effects on B16F10 cells that have been observed at high RSV concentrations. DIRSV reduced melanin synthesis by decreasing tyrosinase activity and downregulating the levels of p-CREB, MITF, and TYR. In addition, it exhibited a strong binding affinity for CREB, along with antioxidant activity in a dose-dependent manner.

RSV, a stilbene-based polyphenolic compound, is recognized for its wide range of biological effects, including antioxidant, anti-inflammatory, and anticancer activities [20,35]. The stilbene structure, comprising a 14-carbon backbone and two phenyl groups connected via an ethene double bond, is responsible for its antioxidant and anti-inflammatory effects [36]. DIRSV and other RSV derivatives, such as those with benzamide, hydroxyl, and hydroxyphenyl groups, exhibit varying antioxidant potencies depending on the chemical modifications that are made to the original compound [37,38,39]. However, when consumed as a dietary component or supplement, RSV is rapidly metabolized in the intestine and liver, primarily by UDP-glucuronosyltransferases and sulfotransferases, which facilitate glucuronidation and sulfation, respectively [40,41]. Following a dietarily relevant 25 mg oral dose of RSV, a study found its plasma concentration to remain in the nanomolar range, while its metabolites were detected in the micromolar range [42,43]. Considering that DIRSV and other derivatives potentially exhibit low bioavailability owing to similar metabolic processes to RSV, this study administered them directly to B16F10 cells to assess their melanogenesis-inhibiting effects.

The current study clearly found DIRSV, as well as RSV, to effectively inhibit melanogenesis by reducing tyrosinase activity. In addition, DIRSV downregulated the protein levels of CREB, p-CREB, MITF, and TYR and demonstrated a strong binding affinity for CREB in silico. Moreover, compared with trans-RSV, DIRSV did not affect cell viability following 24 h treatment at 75 µM or 48 h treatment at 50 µM, suggesting lower cytotoxicity in melanocytes. It has been shown to exhibit at least five-fold lower cytotoxicity, higher solubility, and improved structural stability in mammalian cells [40]. DIRSV is both a plant-derived antimicrobial phytoalexin—primarily isolated from *Dendrobium chrysotoxum*, *Cannabis sativa*, and *Dioscorea dumetorum*—and an RSV metabolite—produced via colonic microbial fermentation—and known for its more stable structure, which is ascribed to the hydrogenation of RSV’s double bond [44,45]. Previous studies have demonstrated several physiological effects of DIRSV, including antioxidant activity in pancreatitis as well as inhibitory effects on adipogenesis and melanogenesis [29,46,47,48]. Our findings are consistent with those of previous studies, which have also reported that DIRSV reduces melanin content, tyrosinase activity, and TRP levels [29]. Furthermore, our study newly discovered that DIRSV’s anti-melanogenic effect may be attributed to its suppression of MITF, a key transcription factor regulating the expression of major melanogenic enzymes (e.g., tyrosinase and TRPs). This effect appears to be mediated by its high binding affinity for CREB and the decreased phosphorylation of CREB.

Although the MITF, TYR, and TRP-1 protein levels decreased, no significant changes in mRNA levels occurred following DIRSV treatment. This may indicate that DIRSV exerts its effects at the post-transcriptional or post-translational level, potentially influencing protein stability or activity without affecting gene expression. Previous studies have revealed that the activation of the extracellular signal-regulated kinase signaling pathway induces MITF phosphorylation at Ser 73, possibly leading to MITF’s ubiquitination and subsequent degradation by the proteasome [49,50]. This potentially accounts for the decrease in MITF and TRP-1 protein levels, while leaving mRNA levels unaffected. Furthermore, the Wnt/β-catenin signaling pathway can regulate the expression of melanogenic genes independently of MITF [51]. β-catenin can translocate to the nucleus and activate MITF, thereby increasing TRP-1 expression. Therefore, the absence of changes in mRNA levels possibly emanates from the regulatory effects of the Wnt/β-catenin pathway.

Based on a previous study reporting that RSV and oxyresveratrol inhibit melanogenesis through their ability to bind to tyrosinase [52], we conducted an in silico molecular docking analysis to evaluate the interaction between RSV derivatives and target proteins that are involved in melanin synthesis. Several recent studies have employed molecular docking analyses to investigate the various functions of RSV by evaluating its affinity for proteins that are involved in these effects, such as the laminin receptor (67LR) in neuroprotection [53] and proteins like “signal transducer and activator of transcription 3” and “mitogen-activated protein kinase” in cardiometabolic benefits [54]. The binding affinities of RSV and its derivatives for tyrosinase or TRP-1 were higher than those of natural compound inhibitors such as tropolone and mimosine, whereas their binding affinities for MITF or CREB were lower compared to those of the known small-molecule inhibitors ML329 and KG-501. However, since all the molecules used in this study share a similar structure with RSV, their affinity values for melanogenesis-related proteins merely displayed minute differences (<0.3 kcal/mol); nonetheless, RSV exhibited the highest binding affinity for tyrosinase compared with DIRSV and the other derivatives. Moreover, RSV, RHS-0139, and RHS-0142 demonstrated the strongest affinity for TRP-1 and MITF among the tested compounds, corroborating its potential role in regulating melanogenesis by inhibiting these key melanogenic enzymes. DIRSV, on the other hand, displayed the highest binding affinity for CREB, suggesting that it may downregulate melanin synthesis via the CREB-mediated signaling pathway, as phosphorylated CREB binds to MITF, enhancing its transcriptional activity [55]. However, since RHS-0139 and RHS-0142, which exhibited high affinity for melanogenesis-related proteins, did not significantly inhibit melanin synthesis, the relationship between binding affinity and functional effects on melanin synthesis warrant further exploration. The actual inhibition of melanin synthesis through the suppression of enzymatic activity appears to involve not only the molecular binding affinity but also a complex interplay of various molecular and cellular regulatory mechanisms.

RSV and DIRSV, in addition to their anti-melanogenic activity, demonstrated concentration-dependent antioxidant potential, as confirmed by both the ABTS and DPPH assays, displaying comparable antioxidant activity to that of Trolox, a water-soluble analog of vitamin E. Furthermore, RSV upregulated *NRF2*, *HO-1*, *GCLc*, *GCLm*, and *NQO1* expression, while DIRSV significantly increased *HO-1*, *GCLC*, and *GCLM* expression in B16F10 cells, confirming the enhancement of antioxidant capacity. GCL, a heterodimer composed of a GLCc and a GCLm, is involved in the de novo synthesis of glutathione, and its expression is tightly regulated by NRF2 [56]. RSV’s antioxidant activity is ascribed to its polyphenolic structure and capacity to activate SIRT1, thus inhibiting mitochondrial superoxide ion production and upregulating antioxidant enzyme expression [57]. Furthermore, previous studies have demonstrated that RSV stimulates NRF2 signaling in human keratinocytes and mouse epidermal cells, increasing glutathione S-transferase activity [57,58,59]. Moreover, RSV also prevents UV-induced skin wrinkles by activating the NRF2/HO-1 pathway, thus preventing photoaging [60]. Similarly, DIRSV has also been reported to quench intracellular ROS, revealing its antioxidant potential [29]. Dietary phenolic compounds reportedly protect against skin pigmentation through their antioxidant and UV-absorbing properties [61,62]. Since UV-induced oxidative stress may serve a central role in the dysregulation of melanogenesis in both melanocytes and melanoma cells, including the inhibition of tyrosinase activity, these compounds may help mitigate its effects [63]. Therefore, the antioxidant effects of RSV and DIRSV may contribute to both skin protection and melanogenic regulation.

The RSV derivatives used in this study—RHS-0139, RHS-0140, RHS-0141, and RHS-0142—are RSV derivatives that contain amide linkers (C=O and NH) between two phenyl groups. Compared to double bonds, amide bonds can form hydrogen bonds. And DIRSV has a high degree of flexibility because it has no double bond. We aimed to identify pharmacophores in the structure of RSV (double bond and hydroxyl groups in A and B ring) by comparing the biological activity of RSV and its derivatives. RSV and DIRSV exhibited similar biological activities. However, the amide derivatives did not show any activity. In particular, RHS-0139 had the same hydroxyl configuration as RSV but did not show any activities. With the introduction of amide bonds, even if they had similar hydroxy groups, they changed to a structure that did not show activity.

Amides, such as salicylamides and N-phenyl benzamide derivatives, have been studied extensively for their biological activities (e.g., antioxidant, anti-inflammatory, and antiviral properties) [64,65,66]. Previous studies have revealed that RSV amide derivatives exhibit inhibitory activity against cyclooxygenase-2 and exert anti-inflammatory effects [66]. In the present study, these compounds exclusively exhibited radical scavenging activity in the ABTS assay, exhibiting no effect in the DPPH assay or on the regulation of Nrf2 and HO-1 expression. Additionally, although they downregulated MITF and CREB levels, they did not impede melanin synthesis or inhibit tyrosinase enzyme activity. These findings suggest that the presence of carbonyl and amine groups between the phenol rings potentially influences the biological activity of RSV derivatives, possibly altering their ability to modulate melanogenesis and antioxidant pathways. Further structural optimization and detailed mechanistic studies are required to more comprehensively elucidate the factors underlying the observed lack of activity in these compounds.

This study has several limitations. First, all in vitro experiments were conducted using only B16F10 murine melanoma cells. While these cells are widely used in melanogenesis research because of their high metastatic potential and natural production of melanin pigment [67,68,69], their relevance to human physiology remains limited. Further studies using human melanocytes, other melanoma cell lines, or 3D skin models are warranted to better reflect clinical applicability. Lastly, while protein expression levels were thoroughly examined, the corresponding mRNA levels did not always show consistent changes, suggesting that post-transcriptional regulation may be involved.

## 4. Materials and Methods

### 4.1. Preparation of RSV and Its Derivatives

RSV derivatives (RHS-0139, RHS-0140, RHS-0141, RHS-0142, and DIRSV) were synthesized using a previously reported method, and their exact molecular structures were confirmed through structural characterization (Figures S1 and S2). Trans-RSV was provided by Suwon University. The samples were dissolved in dimethyl sulfoxide (DMSO, Sigma-Aldrich, St. Louis, MO, USA, D8418). The structures and characteristics of the compounds were verified using ChemSpider (https://www.chemspider.com/ (accessed on 8 November 2024)) and are presented in Table S1.

### 4.2. Cell Culture and Sample Treatment

B16F10 melanoma cells were purchased from the Korea Cell Line Bank (Seoul, Republic of Korea) and cultured in Dulbecco’s modified Eagle medium (DMEM; Gibco, Waltham, MA, USA), supplemented with 10% fetal bovine serum (FBS; Biowest, Nuaillé, France, 078U20) and 1% antibiotic–antimycotic solution (Gibco, L0010-100). Trans-RSV, DIRSV, RHS-0139, RHS-0140, RHS-0141, and RHS-0142, initially dissolved in DMSO at high concentrations, were diluted so that the final concentration of DMSO in the culture medium was 0.05%. To eliminate any solvent-related effects, the same concentration of DMSO was used as for the vehicle control. Trans-RSV was used as a positive control for efficacy evaluation in all experiments.

The cells were maintained in a humidified incubator at 37 °C with 95% air and 5% CO_2_. They were seeded into a six-well plate at a density of 3.4 × 10^4^ cells/well and cultured for 2 days. RSV, DIRSV, RHS-0139, RHS-0140, RHS-0141, and RHS-0142 were subsequently administered at a concentration of 50 µM for 24 h. Thereafter, the cells were collected for further analyses.

### 4.3. Cell Viability

B16F10 cells were seeded in a 96-well plate at a density of 1 × 10^5^ cells/well and cultured for 24 h. RSV, DIRSV, RHS-0139, RHS-0140, RHS-0141, and RHS-0142 were subsequently added at concentrations of 50, 75, and 100 µM for 24 or 48 h. Following treatment, the cell viability was assessed using the 3-(4,5-dimethylthiazol-2-yl)-2,5-diphenyltetrazolium bromide (MTT) assay. The cells were incubated with an MTT reagent at 37 °C for 2 h, after which the reagent was removed. DMSO (Sigma-Aldrich, D8418) was added to dissolve the purple formazan crystals that had formed. Absorbance was measured at 540 nm using a microplate spectrophotometer (Epoch, BioTek Instruments, Winooski, VT, USA).

### 4.4. Measurement of Melanin Content and Tyrosinase Activity

To determine the melanin content, cultured cells were washed twice with phosphate-buffered saline (PBS), collected, and centrifuged at 2000 rpm for 15 min. The resulting pellet was dried, dissolved in 200 µL of 1 N NaOH, and heated at 80 °C for 1 h. Absorbance was measured at 405 nm using a microplate spectrophotometer, and protein normalization was performed using a bicinchoninic acid (BCA) assay kit (Biomax, Seoul, Republic of Korea, BCA0500, BCA0500). To assess the tyrosinase activity, cells were washed with PBS and lysed in 100 µL of 100 mM sodium phosphate (pH 6.8) containing 1% (*w*/*v*) Triton X-100 (Sigma-Aldrich, St. Louis, MO, USA, T9284). After freezing at −80 °C for 30 min, lysates were collected and centrifuged at 2000 rpm for 30 min. A 40 µL aliquot of the supernatant was mixed with 160 µL of 2 mg/mL L-DOPA in 100 mM sodium phosphate buffer (pH 6.8) and incubated at 37 °C for 1 h. Absorbance was measured at 475 nm, and protein normalization was conducted using the BCA assay kit.

### 4.5. Molecular Docking

The protein structures that were used for the molecular docking simulation were obtained from the Research Collaboratory for Structural Bioinformatics (RCSB) Protein Data Bank (https://www.rcsb.org/ (accessed on 3 December 2024)). Four target proteins were analyzed: tyrosinase (PDB ID: 2Y9X), microphthalmia-associated transcription factor (MITF; PDB ID: 4ATI), tyrosinase-related protein 1 (TRP-1; PDB ID: 5M8R), and cAMP response element-binding protein (CREB; PDB ID: 1DH3). The downloaded protein structures were prepared for docking using UCSF Chimeric software (ver. 1.19). The preprocessing steps included the removal of solvent molecules, addition of polar hydrogen, and conversion to the PDBQT file format, which is suitable for docking analysis. Molecular docking simulations were conducted to evaluate the binding affinity of each protein for the small molecules of interest. Binding affinity values (ΔG) were determined to assess the binding potential and stability of the interactions between the proteins and ligands. The docking scores of the natural antagonistic ligands (tropolone and mimosine [70,71]) and small-molecule inhibitors (ML-329 and KG-501 [33,34]), as well as those of the tested ligands (RSV, DIRSV, RHS-0139, RHS-0140, RHS-0141, and RHS-0142), were used as reference values.

### 4.6. Western Blotting

Total proteins were extracted from B16F10 cells using a radioimmunoprecipitation assay buffer (Biomax, Seoul, Republic of Korea, BRA050) supplemented with a protease–phosphate inhibitor cocktail (Thermo Scientific, Waltham, MA, USA). The extracted proteins were separated on sodium dodecyl sulfate–polyacrylamide gels and transferred onto polyvinylidene fluoride membranes. The membranes were blocked and incubated overnight at 4 °C with the following primary antibodies: anti-MITF (1:1000, D5G7V; Cell Signaling, Danvers, MA, USA), anti-TYR (1:200, T311 sc-20035; Santa Cruz Biotechnology, Dallas, TX, USA), anti-TRP-1 (1:200, G-9 sc-166857; Santa Cruz Biotechnology, Dallas, TX, USA), and anti-β-actin (1:2000, D6A8, Cell Signaling). After washing, the membranes were incubated for 1 h at room temperature with a horseradish peroxidase-conjugated rabbit secondary antibody (Cell Signaling, 7074, Danvers, MA, USA) and visualized using West Glow FEMTO chemiluminescent substrate (Biomax, Seoul, Republic of Korea, LOT BWM0C0507). The protein band intensities were quantified using ImageJ software Version 1.54 (National Institutes of Health, Bethesda, MD, USA).

### 4.7. RNA Extraction and Quantitative Real-Time Polymerase Chain Reaction (RT-qPCR)

Total RNA was isolated from B16F10 melanoma cells using RNAiso Plus (Takara, Shiga, Japan). The RNA purity and concentration were measured using a Microvolume Spectrophotometer (DeNovix, Wilmington, DE, USA). Complementary DNA (cDNA) was synthesized from total RNA using the PrimeScript™ II First Strand cDNA Synthesis Kit (Takara, Shiga, Japan). RT-qPCR was performed using the Roche LightCycler^®^ 96 instrument (Roche, Basal, Switzerland) and TB green Premix Ex Taq (Takara, Shiga, Japan). The gene expression levels were normalized to those of *ß-actin*. The primer sequences for *MITF*, *TYR*, *TRP-1*, *TRP-2*, *NRF2*, *HO-1*, and *ß-actin* are listed in Table 2.

### 4.8. Measurement of 1,1-Diphenyl-2-Picrylhydrazyl (DPPH) and 2,2′-Azino-Bis(3-Ethylbenzothiazoline-6-Sulfonic Acid (ABTS)) Radical Scavenging Activity

For the DPPH assay, a working solution of DPPH (Sigma-Aldrich, St. Louis, MO, USA, D9132) was dissolved in ethanol, and the absorbance was measured at 515 nm. RSV, DIRSV, RHS-0139, RHS-0140, RHS-0141, RHS-0142, and vitamin C (1 mg/dL, standard) were tested at 200, 400, and 800 µM. Each sample (20 µL) was mixed with 380 µL of DPPH solution and incubated in the dark for 30 min at room temperature. The absorbance was measured at 515 nm, and the DPPH radical scavenging activity was calculated. For the ABTS assay, ABTS (Sigma-Aldrich, St. Louis, MO, USA, A9941) was prepared by mixing 7 mM ABTS with 2.45 mM potassium persulfate (1:1) and incubating the resulting mixture in the dark to stabilize it. The solution was adjusted to an absorbance of 0.8–1.2 at 734 nm with PBS. The samples (200, 400, and 800 µM) were mixed with Trolox (5 mg/mL, standard) and ABTS, and the resulting mixture was incubated in the dark for 10 min at room temperature. The absorbance was measured at 734 nm, and the ABTS radical scavenging activity was calculated.

$$\mathrm{DPPH}\;\mathrm{or}\;\mathrm{ABTS}\;\mathrm{radical}\;\mathrm{scavenging}\;\mathrm{activity}\;(\%)=(\frac{{ABS}_{control-}{ABS}_{sample}}{{ABS}_{control}})\times 100$$

### 4.9. Statistical Analysis

All results are presented as the mean ± standard error (SE). Statistical differences between experimental groups were evaluated using one-way ANOVA. When a significant F value was observed (*p* < 0.05), a Least Significant Difference (LSD) post hoc test between two groups was subsequently performed to determine pairwise group differences. A *p*-value < 0.05 was considered statistically significant. All statistical analyses were performed using SPSS (version 26; IBM SPSS Inc., Chicago, IL, USA).

## 5. Conclusions

This study confirms that DIRSV, like RSV, inhibits melanogenesis and exhibits antioxidant effects in B16F10 cells, demonstrating its potential as a skin-protective and skin-whitening agent. In contrast, RSV amide derivatives exhibit partial antioxidant activity and inhibit melanogenesis-related proteins but do not significantly affect melanin synthesis. Since DIRSV does not diminish cell viability compared with RSV, it holds potential as a whitening agent. To explore its practical applications and enhance the activity of other derivatives, further research is warranted.

## Figures and Tables

**Table 1:** Degree of expression through ligand (RSV and RSV derivatives)–target protein interaction.

	Affinity with Protein (kcal/mol)			
Ligand	Tyrosinase	MITF	TRP-1	CREB
Resveratrol	−7.3	−5.1	−7.5	−5.2
Dihydroresveratrol	−7.0	−4.9	−7.2	−5.3
RHS-0139	−7.1	−5.1	−7.5	−5.1
RHS-0140	−7.1	−5.0	−7.2	−5.3
RHS-0141	−7.0	−5.1	−7.7	−5.1
RHS-0142	−7.1	−5.1	−7.5	−5.2
Tropolone	−5.9	-	-	-
ML329	-	−5.8	-	-
Mimosine	-	-	−6.2	-
KG-501	-	-	-	−6.1

The binding affinity is expressed as an energy value, and the higher the value is, the greater the binding force is. Tropolone is used as the tyrosinase antagonist, ML329 as the MITF antagonist, mimosine as the TRP-1 antagonist, and KG-501 as the CREB antagonist.

**Table 2:** Primer sequences used for real-time qPCR analyses in B16F10 melanoma cell.

Gene	Forward Primer (5′→3′)	Reverse Primer (5′→3′)
Mouse *MITF*	TTCCGTTACCTTACCCAGAGG	AGACGCAGTGTTTTTGCTCAC
Mouse *TYR*	TTAGGATTTTCAGGGTGACGAC	TGGAGGGACATTGATTTTGC
Mouse *TRP-1*	TATTGGCACACTCTCGTGGA	CATCTGAGCACCCCTGTCTT
Mouse *TRP-2*	AAGTTGCTCTGCGGTTAGGA	AACGACCCTGTGTTTGTGGT
Mouse *NRF2*	GTCACTGGGCTCTGCTATGAA	TCTCCTCGCTGGAAAAAGAA
Mouse *HO-1*	GGTGAGGGAACTGTGTCAGG	CAGGGGCTGTGAACTCTGTC
Mouse *GCLC*	GAGAGCCTGATGTTCGCCTA	GAGAAGGGGGAGAGGACAAA
Mouse *GCLM*	TTGGAGTTGCACAGCTGGATT	TGGTTTTACCTGTGCCCACTG
Mouse *NQO1*	GACCTTGCTTTCCATCACCACCGG	GTAGAGTGGTGACTCCTCCCAGAC
Mouse *ß-actin*	AATCGTGCGTGACATCAA	GCTCGTTGCCAATAGTGA

## Data Availability

The original contributions presented in this study are included in the article. Further inquiries can be directed to the corresponding authors.

## Supplementary Materials

The following supporting information can be downloaded at https://www.mdpi.com/article/10.3390/ijms26114999/s1.

## Abbreviations

ABTS	2,2′-azinobis-(3-ethylbenzothiazoline-6-sulphonic acid)
cAMP	Cyclic Adenosine Monophosphate
CREB	cAMP response element-binding protein
DIRSV	Dihydroresveratrol
DMSO	Dimethyl sulfoxide
DPPH	2,2-Diphenyl-1-picrylhydrazyl
GCLC	Glutamate-cysteine ligase catalytic subunit
GCLM	Glutamate-cysteine ligase regulatory subunit
H_2_O_2_	hydrogen peroxide
HO-1	Heme Oxygenase-1
MTT	3-(4,5-dimethylthiazol-2-yl)-2,5-diphenyltetrazolium bromide
MAPK	mitogen-activated protein kinase
MC1R	Melanocortin 1 receptor
MITF	Microphthalmia-associated transcription factor
NRF2	Nuclear factor erythroid 2-related factor 2
NQO1	NAD(P)H dehydrogenase 1
p-CREB	Phosphorylated CREB
PBS	Phosphate-buffered saline
PKA	Protein Kinase A
ROS	Reactive oxygen species
RSV	Resveratrol
SIRT1	Sirtuin 1
TRP-1	Tyrosinase related protein-1
TRP-2	Tyrosinase related protein-2
TRPs	Tyrosinase Related Proteins
UV	ultraviolet
Wnt/β-catenin	Wnt/β-catenin Signaling Pathway
α-MSH	α-Melanocyte-stimulating hormone
β-catenin	catenin beta-1
