# Supplementary material for: Effects of Resveratrol Derivatives on Melanogenesis and Antioxidant Activity in B16F10 Cells
Source: Int J Mol Sci. 2025 May 22;26(11):4999. doi: 10.3390/ijms26114999 (PMC12154001; doi:10.3390/ijms26114999)
Supplement: Supplementary file 1 [file ijms-26-04999-s001.zip › ijms-3605526-supplementary.pdf]

Supplementary files

A. ^{1}H NMR Spectra for Dihydroresveratrol

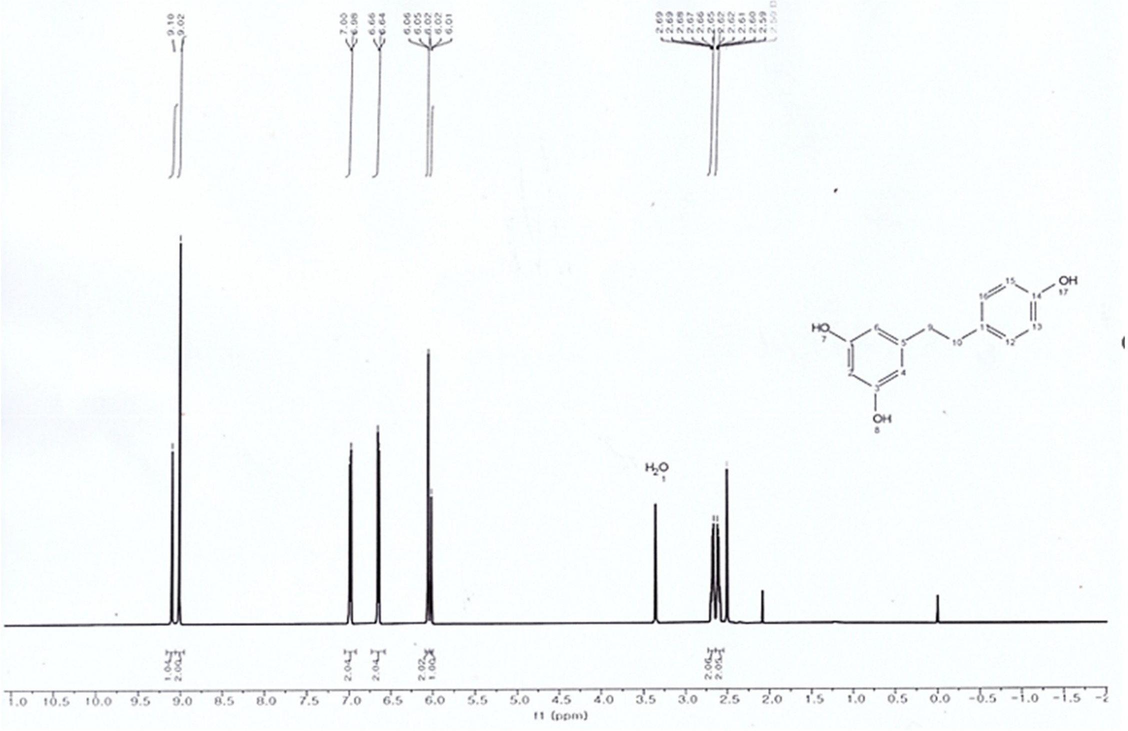

^{1}H NMR (300 MHz, DMSO-d_6) δ 6.39 (s, 1H), 6.481 (d, 1H, J = 7.5 Hz), 6.73 (s, 2H), 7.09 (m, 2H), 7.33 (s, 1H), 9.39 (bs, 1H), 9.53 (bs, 2H), 9.93 (s, 1H).

B. ^{1}H NMR Spectra for RHS-0139

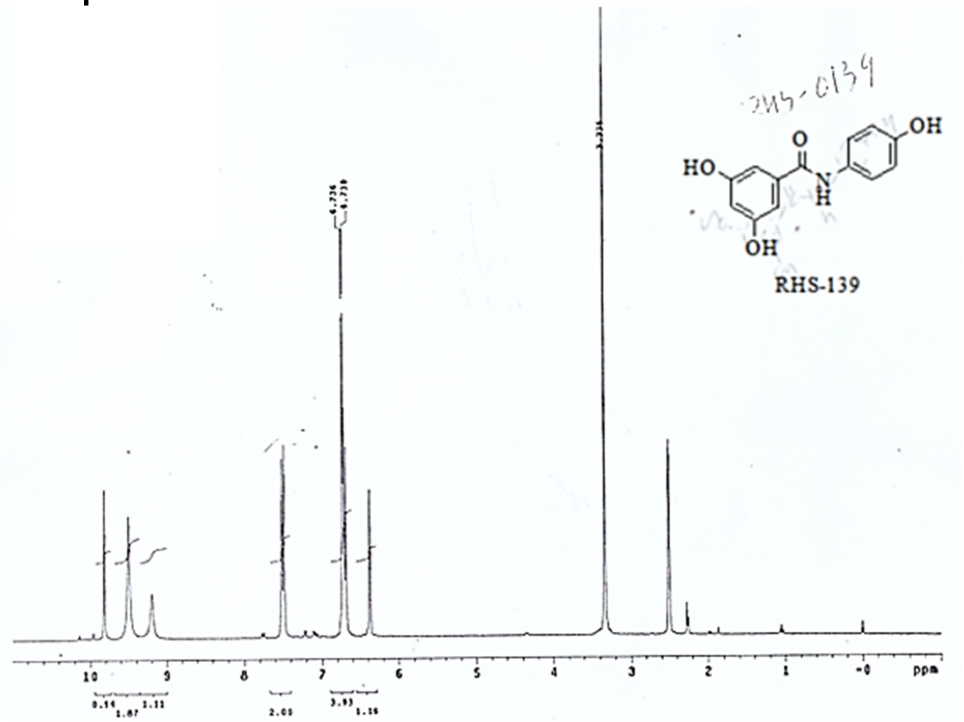

^{1}H NMR (300 MHz, DMSO-d_6) δ 6.25 (s, 1H), 6.62 (m, 4H), 4.42 (d, 2H, J = 8.7 Hz), 9.19 (s, 1H), 9.42 (bs, 2H), 9.81 (s, 1H).

C. ¹H NMR Spectra for RHS-0140

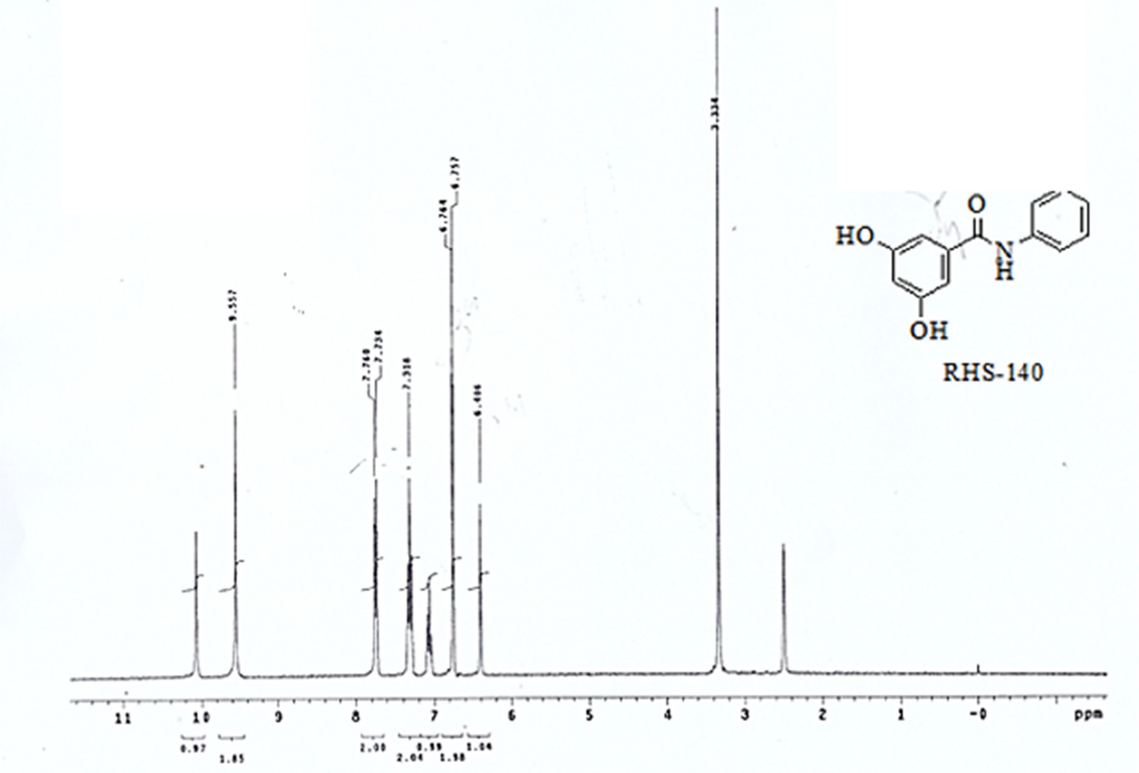

^{1}H NMR (300 MHz, DMSO-d$_6$) δ 6.40 (s, 1H), 6.76 (s,2H), 7.03 (m, 1H), 7.31 (m, 2H), 7.76 (d, 2H, J = 7.8 Hz), 9.55 (s, 2H), 10.02 (s, 1H).

D. ¹H NMR Spectra for RHS-0141

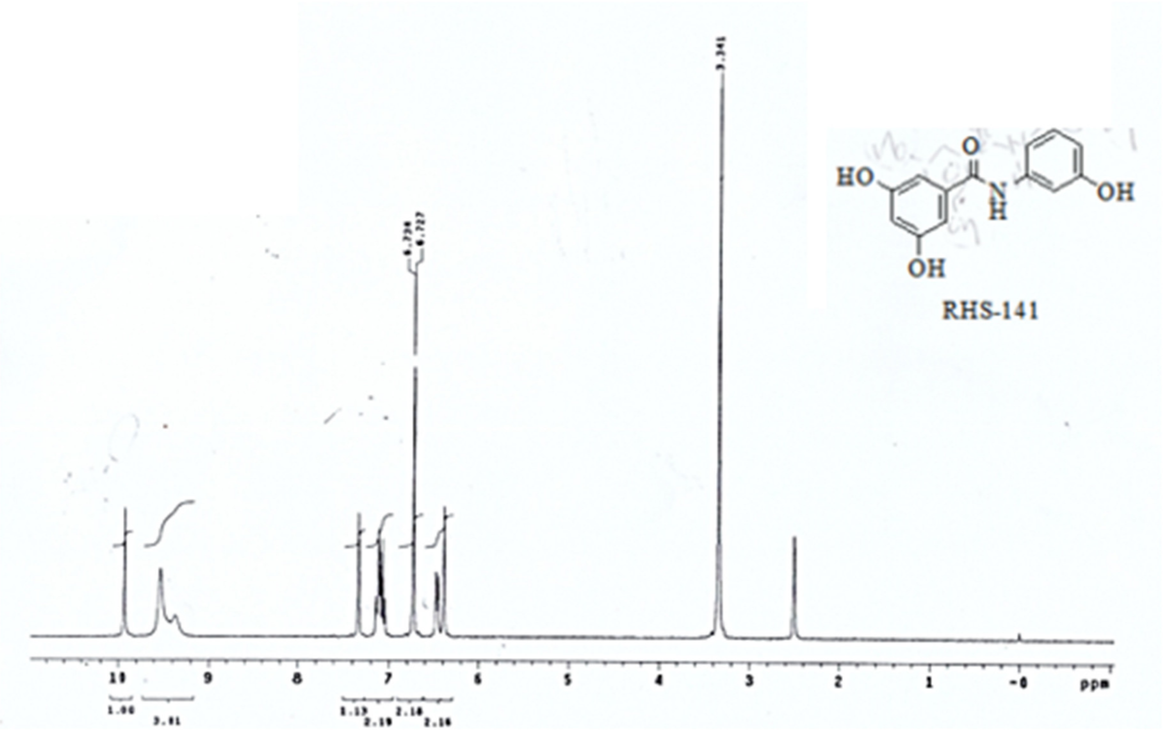

^{1}H NMR (300 MHz, DMSO-d$_6$) δ 6.39 (s, 1H), 6.481 (d, 1H, J = 7.5 Hz), 6.73 (s, 2H), 7.09 (m, 2H), 7.33 (s, 1H), 9.39 (bs, 1H), 9.53 (bs, 2H), 9.93 (s, 1H).

E. ^{1}H NMR Spectra for RHS-0142

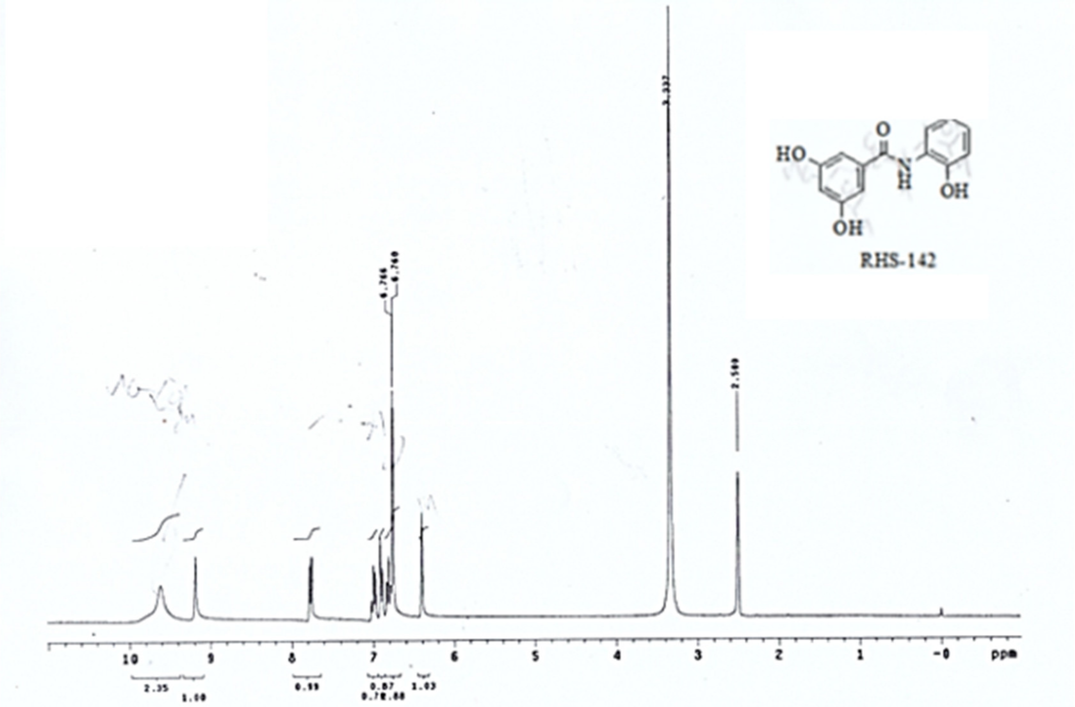

^{1}H NMR (300 MHz, DMSO-d_6) δ 6.40 (s, 1H), 7.76 (s, 2H), 6.81 (m, 1H), 6.91 (m, 1H), 7.02 (m, 2H), 7.75 (d, 1H, J = 7.5 Hz), 9.19 9s, 1H), 9.70 (bs, 3H).

Figure S1. ^{1}H NMR Spectra for resveratrol derivatives (A) Dihydroresveratrol, (B) RHS-0139, (C) RHS-0140, (D) RHS-0141, and (E) RHS-0142. ^{1}H NMR spectra were recorded in DMSO-d_6 on a 300 MHz Mercury-300 (Varian) spectrometer with TMS as the internal standard.

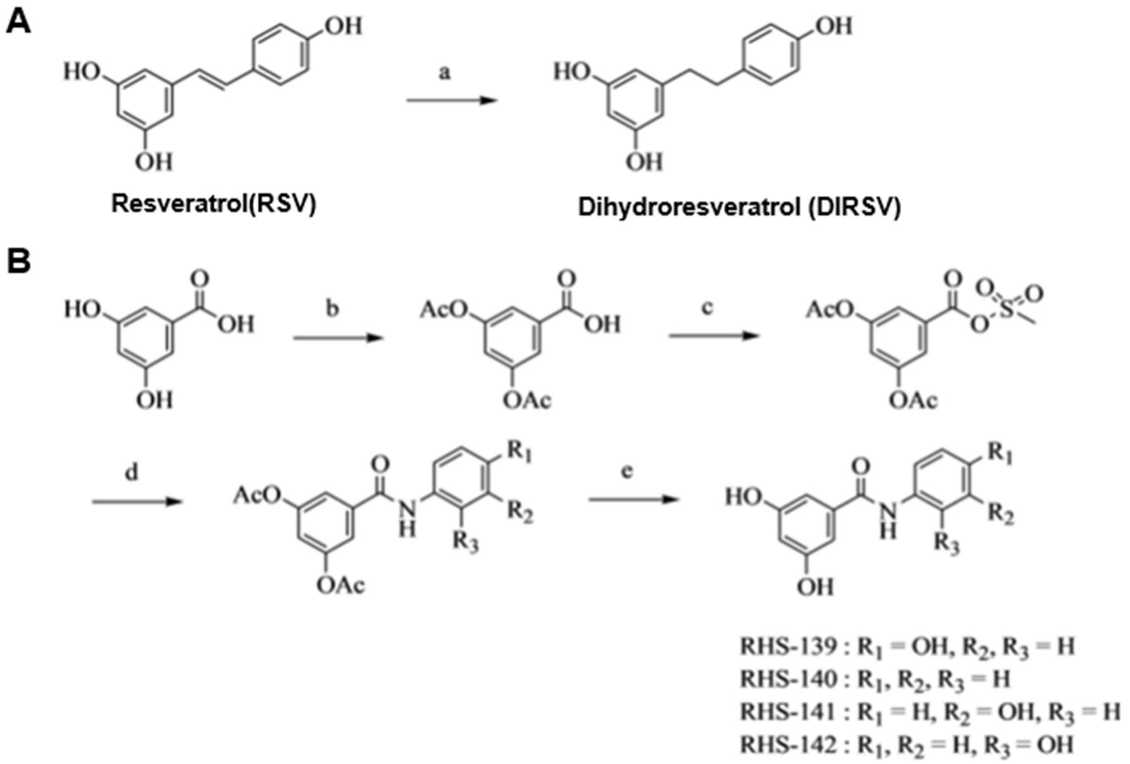

Figure S2. Synthesis of resveratrol derivatives (A) Synthesis of dihydroresveratrol: (a) Pd/C, H_2, 1atm, (B) Synthesis of amide derivatives: (b) acetic anhydride, triethylamine, tetrahydrofuran, (c) $MeSO_2$-Cl, triethylamine, tetrahydrofuran, (d) Aniline, triethylamine, tetrahydrofuran, (e) KOH, H_2O

Table S1. Structure of resveratrol and its derivatives.

Name	Molecular formula	Chem spider ID	SMILES
Trans-resveratrol	C14H12O3	392875	c1cc(ccc1/C=C/c2cc(cc(c2)O)O)O[1)2)3)]
Dihydro-resveratrol	C14H14O3	161607	c1cc(ccc1CCc2cc(cc(c2)O)O)O[4)]
Hydroxyphenyl Dihydroxybenzamide (RHS-0139)	C13H11NO4	28575553	c1cc(ccc1NC(=O)c2cc(cc(c2)O)O)O[5)]
3,5-Dihydroxy-N-phenylbenzamide (RHS-0140)	C13H11NO3	49866829	c1ccc(cc1)NC(=O)c2cc(cc(c2)O)O
3,5-Dihydroxy-N-(3-hydroxyphenyl)benzamide (RHS-0141)	C13H11NO4	35787083	c1cc(cc(c1)O)NC(=O)c2cc(cc(c2)O)O
3,5-Dihydroxy-N-(2-hydroxyphenyl)benzamide (RHS-0141)	C13H11NO4	59442003	c1ccc(c(c1)NC(=O)c2cc(cc(c2)O)O)O

1) c1 : c1 : Refers to a benzene ring (aromatic ring).
2) cc : Double bond between carbons in the aromatic ring.
3) /C=C/ : Carbon chain with a double bond, indicating E-stereochemical configuration.
4) c2cc(cc(c2)O)O : Catechol structure with two hydroxyl groups attached. 5) NC(=O) : Amide group (-NHCO)
